# Development and Optimization of a Molecularly Imprinted Polymer for Electrochemical Detection of *Escherichia coli*

**DOI:** 10.3390/polym18202508

**Published:** 2026-10-14

**Authors:** Chuchart Pintavirooj, Naphatsawan Vongmanee, Sarinporn Visitsattapongse

**Affiliations:** 1 Department of Biomedical Engineering, School of Engineering, King Mongkut’s Institute of Technology Ladkrabang, Bangkok 10520, Thailand; 2 Department of Physics, Faculty of Science, King Mongkut’s University of Technology Thonburi, Bangkok 10140, Thailand; naphatsawan.vong@kmutt.ac.th

**Keywords:** *Escherichia coli*, electrochemical sensor, molecularly imprinted polymer, screen-printed electrode

## Abstract

In this study, a molecularly imprinted polymer (MIP)-based electrochemical biosensor was developed for the selective detection of *Escherichia coli* (*E. coli*). The MIP recognition layer was synthesized by free-radical polymerization using MAM, AAM and VP as functional monomers with *E. coli* cells as the imprinting template. The MIP was immobilized onto a screen-printed electrode and evaluated using cyclic voltammetry (CV). Six MIP formulations with different MAM:AAM ratios were investigated to determine the optimal monomer composition. The optimized formulation, with an MAM:AAM ratio of 2:3:1, was evaluated over an *E. coli* concentration range of 10–10^5^ CFU/mL and exhibited a linear response with a slope of 4.1107 and an R^2^ value of 0.9893 with a detection limit of 1.52 CFU/mL. The selectivity of the optimized sensor was further evaluated using *Enterococcus faecalis* and *Pseudomonas aeruginosa*, which showed lower responses than *E. coli*. These results indicate that the proposed MIP-based electrochemical biosensor provides preferential recognition of *E. coli* under the investigated conditions and demonstrates potential for bacterial detection.

## 1. Introduction

*Escherichia coli* (*E. coli*) is a Gram-negative bacterium that is commonly found in the intestinal tract of humans [1]. Although most strains are harmless and contribute to maintaining the normal intestinal microbiota [2], several pathogenic strains are responsible for a wide range of infectious diseases, including urinary tract infections, gastroenteritis, septicemia, neonatal meningitis, and foodborne illnesses [3,4,5,6,7]. Moreover, *E. coli* is widely recognized as an important indicator of fecal contamination in environmental water and food products [8]. Consequently, rapid detection of *E. coli* is essential for clinical diagnosis, food safety monitoring, and environmental surveillance [9,10].

Conventional methods for *E. coli* detection mainly include bacterial culture, Gram staining, biochemical identification, enzyme-linked immunosorbent assay (ELISA) and polymerase chain reaction (PCR) [11,12,13,14]. Although these techniques generally provide satisfactory sensitivity and specificity, they often require labor-intensive sample preparation, sophisticated instrumentation, trained personnel, or prolonged analysis times [15,16]. For instance, bacterial culture, which remains the gold standard for bacterial identification, typically requires 24–72 h before results become available [17]. Molecular techniques such as PCR offer excellent analytical sensitivity but rely on expensive instrumentation and reagents, limiting their applicability in routine laboratories and point-of-care testing [18]. Therefore, there is an increasing demand for rapid, simple, and cost-effective analytical platforms capable of detecting bacterial pathogens with high sensitivity and selectivity.

Electrochemical biosensors have attracted considerable attention as alternative analytical platforms because of their rapid response, high sensitivity, low sample consumption, ease of operation and compatibility with miniaturized and portable devices [19]. Electrochemical techniques, including cyclic voltammetry (CV), differential pulse voltammetry (DPV), square wave voltammetry (SWV), and electrochemical impedance spectroscopy (EIS), have been extensively employed for bacterial detection [20,21,22,23,24]. Among these methods, CV is particularly useful for investigating electron-transfer behavior at the electrode surface and monitoring electrochemical signal changes resulting from bacterial recognition [25,26]. In combination with screen-printed electrodes (SPEs), electrochemical sensing platforms offer additional advantages such as low fabrication cost, disposability and suitability for large-scale production, making them attractive candidates for rapid bacterial detection [27,28]. Conductive polymers have attracted considerable attention in electrochemical applications due to their electrical conductivity and redox activity, which facilitate electron transfer and electrochemical signal transduction. Their favorable electrochemical properties make them useful for various applications, including drug delivery and sensing. For example, PEDOT-based composite films have been investigated for electrochemical drug-release applications, demonstrating the potential of conductive polymers in electrochemical systems [24].

Previous studies have demonstrated the feasibility of bacterial MIP-based electrochemical sensors for *E. coli* using different recognition strategies and functional monomers. For example, boronic-acid-based MIPs have been employed to recognize carbohydrate-containing structures on the *E. coli* surface, while polydopamine-based bacterial imprinted films and other functional polymer systems have also been developed to improve bacterial recognition and electrochemical response [29]. However, these studies have primarily focused on developing specific MIP architectures or improving electrode configurations and sensing performance, whereas the systematic evaluation of how the relative composition of multiple functional monomers affects bacterial recognition remains less explored. In particular, a comparative investigation of methacrylamide (MAM), acrylamide (AAM), and N-vinylpyrrolidone (VP) within the same MIP platform for *E. coli* detection has not been sufficiently established. Therefore, systematically optimizing the MAM:AAM:VP composition may provide useful insight into the relationship between functional-monomer composition, bacterial recognition, and electrochemical response.

The analytical performance of electrochemical biosensors is determined by the recognition element immobilized on the electrode surface [30]. Conventional biological recognition elements, including antibodies, enzymes, and nucleic acid aptamers, exhibit excellent affinity and specificity toward their target analytes [31,32]. However, these biomolecules often suffer from limited chemical stability, high production costs, complex immobilization procedures, and susceptibility to environmental conditions such as temperature and pH. These limitations have motivated the development of synthetic recognition materials that offer improved stability, lower production costs, and longer operational lifetimes.

Molecularly imprinted polymers (MIPs) have emerged as promising synthetic recognition materials because of their excellent chemical and mechanical stability, low fabrication cost, ease of preparation, and long-term storage stability [33]. MIPs are synthesized by polymerizing functional monomers in the presence of a template molecule or microorganism [34]. After removal of the template, complementary cavities possessing specific size, shape and chemical functionalities remain within the polymer matrix, enabling selective rebinding of the target analyte [35]. Compared with biological recognition elements, MIPs exhibit superior robustness and can withstand harsh chemical environments, making them particularly attractive for electrochemical sensing applications [36]. Recent studies have demonstrated the successful application of MIPs for detecting proteins, small molecules, viruses, and bacterial cells [37,38,39,40]. Nevertheless, the analytical performance of bacterial MIPs is highly dependent on the interaction between functional monomers and bacterial surface components [41]. Systematic investigations on the influence of functional monomer selection for improving the recognition efficiency of *E. coli* MIPs remain relatively limited, highlighting the need for further optimization of polymer composition.

The interaction between functional monomers and bacterial surface biomolecules plays a crucial role in determining the selectivity and binding efficiency of MIPs [42,43]. Appropriate selection of functional monomers can significantly enhance the formation of recognition cavities and improve target rebinding. In this study, MAM, AAM and VP were selected as functional monomers because they contain polar functional groups capable of interacting with bacterial surface components through hydrogen bonding and dipole–dipole interactions [44]. MAM and AAM contain amide groups that facilitate intermolecular interactions with bacterial membrane proteins and polysaccharides, whereas VP contains a lactam group that can participate in hydrogen-bonding interactions and improve the hydrophilicity of the matrix [45,46]. To provide structural stability to the imprinted polymer network, N,N’-(1,2-dihydroxyethylene)bisacrylamide (DHEBA) was employed as the crosslinking agent, while 2,2′-azobis(2-methylpropionitrile) (AIBN) served as the free-radical initiator during UV-induced polymerization [47].

Therefore, this study aimed to develop a molecularly imprinted polymer-based electrochemical sensor for the selective detection of *E. coli* using screen-printed electrodes as the sensing platform. Rather than focusing solely on sensor fabrication, this study systematically investigated the effect of functional-monomer composition on bacterial recognition and electrochemical sensing performance. Different MAM:AAM:VP compositions were evaluated to identify an optimal polymer formulation and to clarify the relationship between monomer composition and analytical response to *E. coli*. The fabricated MIP sensors were characterized by cyclic voltammetry, and their sensitivity, selectivity, and linearity were evaluated. The resulting comparison provides material-design insight into the role of functional-monomer composition in bacterial MIP formation and may support the rational design of MIP-based electrochemical sensors for bacterial detection.

## 2. Materials and Methods

### 2.1. Bacteria Preparation

*E. coli* from the stock culture maintained at the Laboratory of Biomedical Engineering, King Mongkut’s Institute of Technology Ladkrabang, Bangkok, Thailand, was inoculated into Luria–Bertani (LB) broth (Merck, Darmstadt, Germany) and incubated at 37 °C for 16 h [48]. The bacterial suspension was subsequently subjected to a tenfold serial dilution. The diluted samples were plated using the spread plate technique and incubated to allow colony formation. After incubation, bacterial colonies were observed on the LB agar plates (Merck, Darmstadt, Germany) and counted to calculate the number of colony-forming units (CFU/mL). The initial bacterial concentration was calculated to be 7.8 × 10^8^ CFU/mL, which was subsequently used to prepare the desired bacterial concentrations in the following experiments.

### 2.2. Bacteria Fixation

The bacterial stock suspension was washed three times with phosphate-buffered saline (PBS) (P4417, Sigma-Aldrich, Darmstadt, Germany) to remove residual nutrients from the LB broth. The fixation and dehydration procedures were based on a previously reported protocol. The bacterial cells were then fixed with 2.5% glutaraldehyde (G5882, Sigma-Aldrich, Darmstadt, Germany) for 2.5 h at room temperature. Following fixation, the cells were sequentially dehydrated in 50%, 60%, 70%, 80%, and 90% ethanol (459844, Sigma-Aldrich, St. Louis, MO, USA) for 15 min at each concentration and subsequently treated twice with 100% ethanol for 15 min per treatment. After dehydration, the bacterial cells were washed three times with deionized (DI) water to remove residual ethanol and finally resuspended in PBS for subsequent experiments.

### 2.3. Polymer Synthesis for E. coli Detection

Molecularly imprinted polymers (MIPs) for *E. coli* detection were synthesized using methacrylamide (MAM) (109606, Aldrich, St. Louis, MO, USA), acrylamide (AAM) (A9099, Sigma-Aldrich, St. Louis, MO, USA), and N-vinylpyrrolidone (VP) (808518, Sigma-Aldrich, St. Louis, MO, USA) as functional monomers [21,25]. Six polymer formulations were prepared by maintaining the VP molar proportion at 1 while varying the relative molar proportions of MAM and AAM, as summarized in Table 1.

The prepolymer preparation was adapted from previously reported procedures [21,25]. For each formulation, 47 mg of DHEBA (294381, Aldrich, St. Louis, MO, USA) was added as the crosslinking agent to enhance the mechanical stability of the polymer network. In addition, 1.5 mg of AIBN was used as the free-radical initiator. Dimethyl sulfoxide (DMSO) at 300 µL was added to each formulation, and the mixture was thoroughly homogenized. The resulting solution was heated at 80 °C for 10 min to initiate polymerization and form a partially polymerized prepolymer solution for subsequent electrode fabrication [49].

### 2.4. Fabrication of the MIP-Modified Electrode

**Figure 1:**
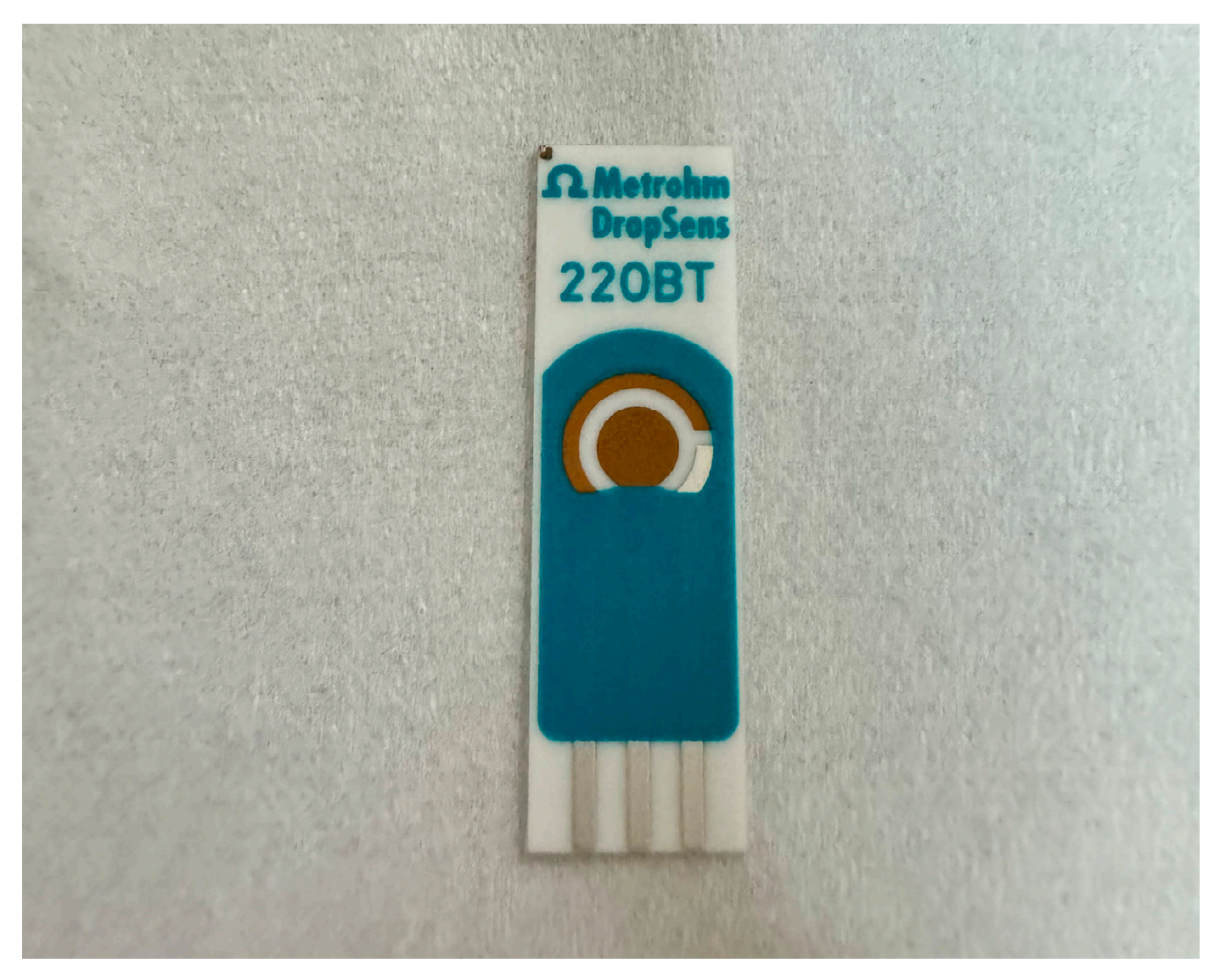
Screen-printed gold electrode used for electrochemical measurements.

A screen-printed electrode (SPE, DRP-220BT, Metrohm DropSens, Llanera, Spain) was used as the sensing platform. The SPE consisted of a gold working electrode (WE), a gold counter electrode (CE), and a silver reference electrode (RE). The configuration of the SPE is shown in Figure 1.

The prepolymer gels prepared under the six synthesis conditions were separately coated onto the working electrode surfaces. For each electrode, 1 μL of the partially polymerized prepolymer solution was applied to the working electrode surface, followed by deposition of 1 μL of fixed *E. coli* suspension at 7.8 × 10^8^ CFU/mL onto each prepolymer layer. The coated electrode was then exposed to UVA light at 365 nm for 3 h to initiate pre-polymerization. The distance between the UVA lamp and the electrode surface was approximately 30 cm. Subsequently, the electrode was incubated in a hot-air oven at 60 °C for 24 h to promote further polymerization and stabilization of the polymer network around the bacterial template. This sequential polymerization procedure was designed to maintain the workability of the prepolymer during template deposition while allowing the polymer network to be further formed after template incorporation.

Following polymerization, template removal was performed by immersing the electrodes in 10% (*v*/*v*) acetic acid for 30 min and then rinsing them with deionized (DI) water for 30 min to eliminate residual acetic acid and detached bacterial cells [21]. The resulting molecularly imprinted polymer (MIP)-modified electrodes were subsequently used for electrochemical measurements.

### 2.5. Electrochemical Measurements for E. coli Detection

Before electrochemical testing, *E. coli* suspensions were prepared at concentrations ranging from 10 to 10^5^ CFU/mL in a ferri/ferrocyanide redox couple solution. Each bacterial suspension was then applied to the MIP-modified electrode. Cyclic voltammetry measurements were performed using a potentiostat over a potential range of −0.3 to +0.6 V at a scan rate of 50 mV s^−1^. The current change was calculated as ΔI = I_blank_ − I_sample_, and the percentage current change was calculated as (ΔI/I_blank_) × 100.

## 3. Results

**Figure 2:**
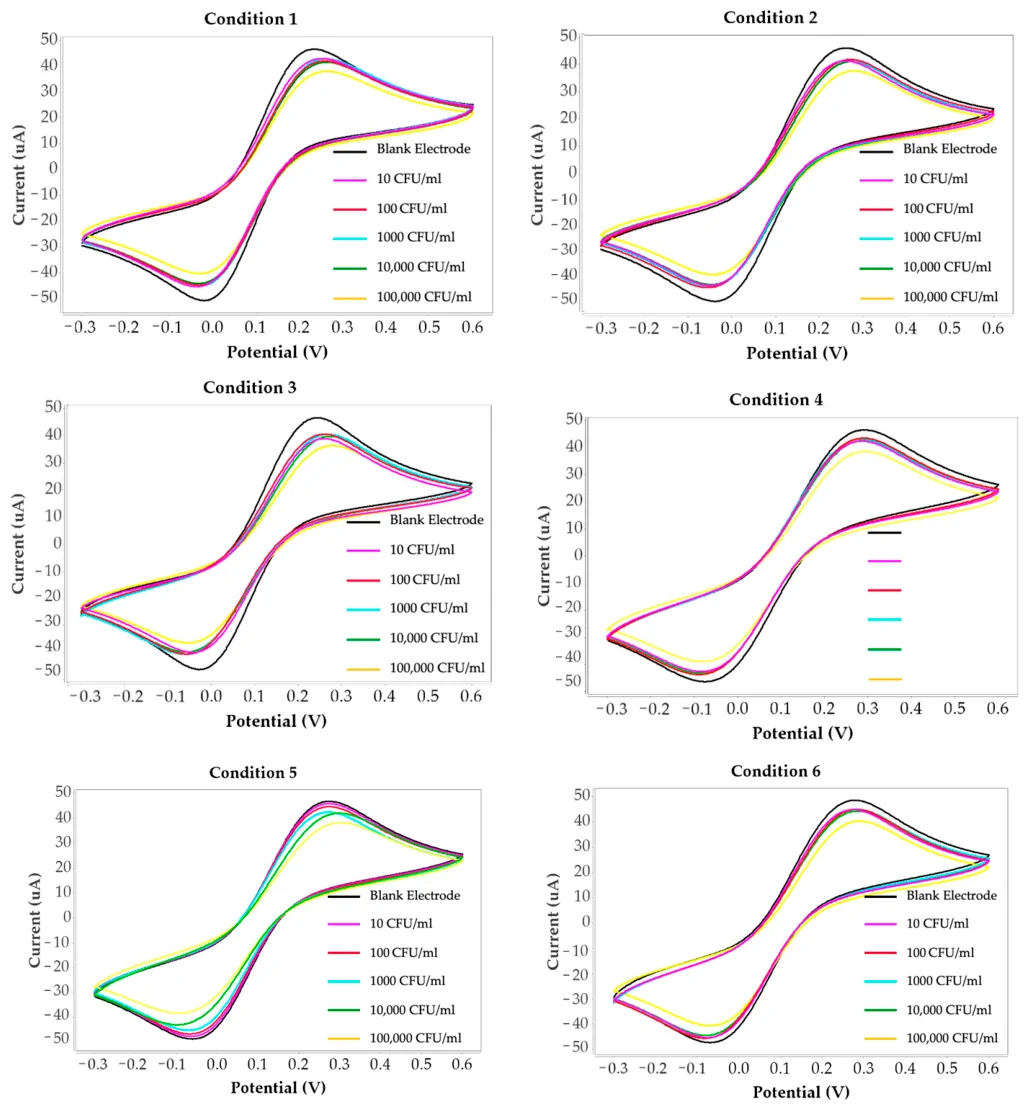
Cyclic voltammograms obtained under six different polymer synthesis conditions at a scan rate of 50 mV/s. The MAM:AAM:VP ratios were 1:1:1 (Condition 1), 2:1:1 (Condition 2), 1:2:1 (Condition 3), 3:2:1 (Condition 4), 2:3:1 (Condition 5) and 3:3:1 (Condition 6).

**Figure 3:**
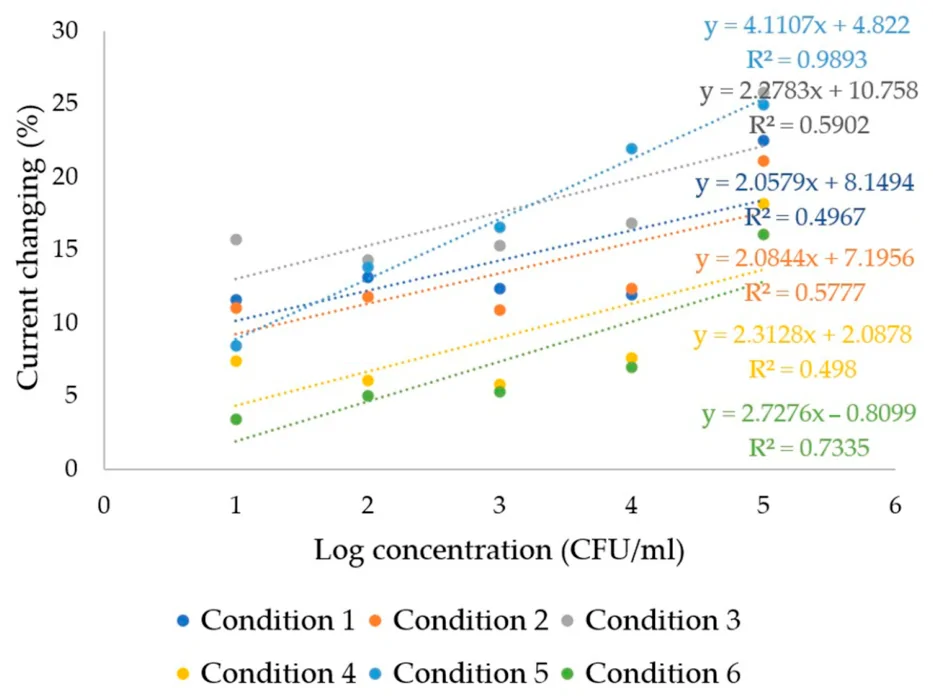
Linear comparison of the current responses obtained under six different polymer synthesis conditions.

The cyclic voltammograms obtained for *E. coli* detection under six different polymer synthesis conditions are shown in Figure 2 and the corresponding current responses are presented in Table 2. The linear relationships between the percentage current change and the logarithmic *E. coli* concentration for the six polymer synthesis conditions are shown in Figure 3.

The results demonstrated differences in electrochemical responses among the six polymer synthesis conditions, indicating that 2:3:1 (Condition 5) was optimal. This condition exhibited a slope of 4.1107 and an R^2^ value of 0.9893, indicating a good linear relationship between the *E. coli* concentration and the electrochemical response. Based on the slope and R^2^ values, polymer synthesis condition 5 was selected for subsequent selectivity evaluation.

The electrochemical response was evaluated using CV with ferri/ferrocyanide as the redox probe. The observed changes in peak current were attributed to changes in the accessibility of the electrode surface to the redox probe. Before bacterial rebinding, the redox probe can readily reach the electrode surface and undergo electron transfer, resulting in a higher peak current. Following bacterial rebinding to the imprinted recognition sites, the bacterial cells partially block the electrode surface and hinder the diffusion of the redox probe toward the electrode, resulting in a decrease in the peak current.

The sensitivity of the sensor varied with the polymer composition because the monomer ratio affects the formation and availability of the molecularly imprinted recognition sites. An appropriate ratio of functional monomers is expected to provide a suitable balance between template–monomer interactions and the accessibility of the resulting recognition sites. This can facilitate bacterial rebinding and produce a more pronounced change in the ferri/ferrocyanide redox current. In contrast, non-optimal monomer ratios may result in less favorable recognition-site formation or reduced accessibility of the binding sites, leading to a weaker electrochemical response.

The irregular changes in current observed at some bacterial concentrations may be attributed to variations in bacterial rebinding and the heterogeneous blocking of the electrode surface. Because bacterial cells are relatively large compared with the molecular recognition sites, their binding can produce non-uniform coverage of the electrode surface. Therefore, the current response may not change monotonically at every individual concentration, particularly during the preliminary screening of different polymer compositions. The overall concentration-dependent response and linear regression obtained for the optimized polymer condition were therefore used to evaluate the analytical performance of the sensor.

**Figure 4:**
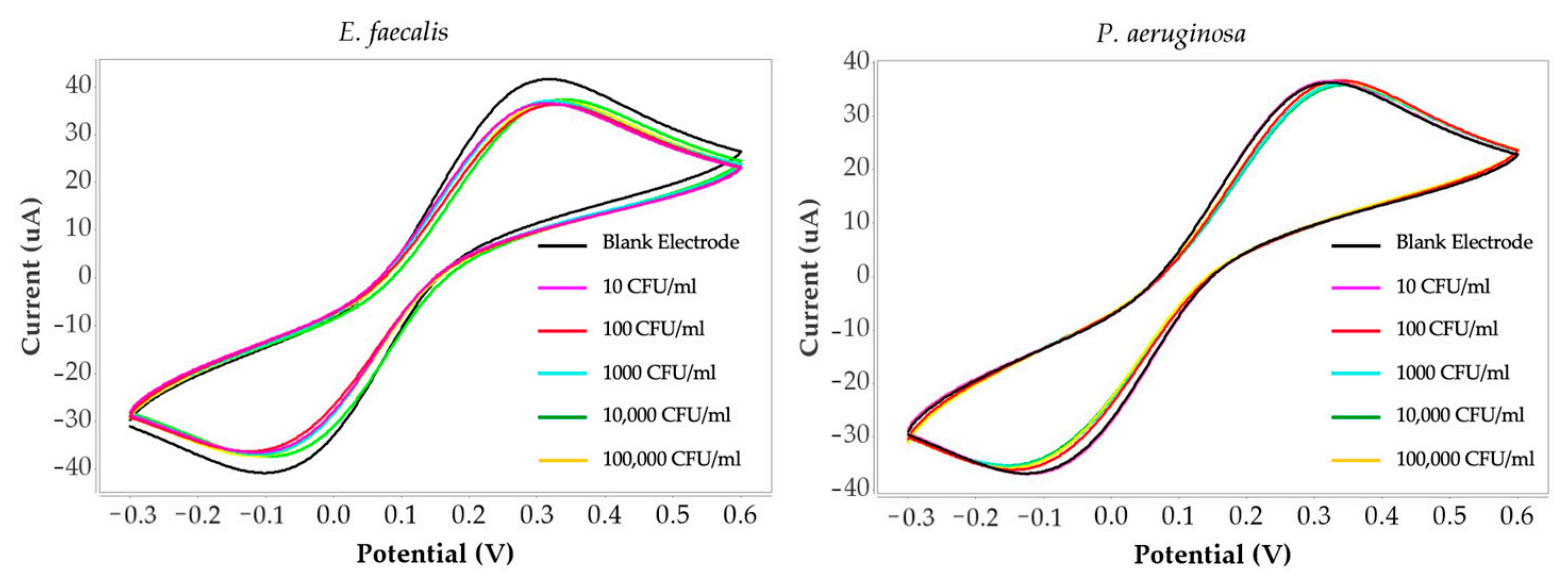
Cyclic voltammograms obtained for non-target bacteria, *E. faecalis* and *P. aeruginosa*.

**Figure 5:**
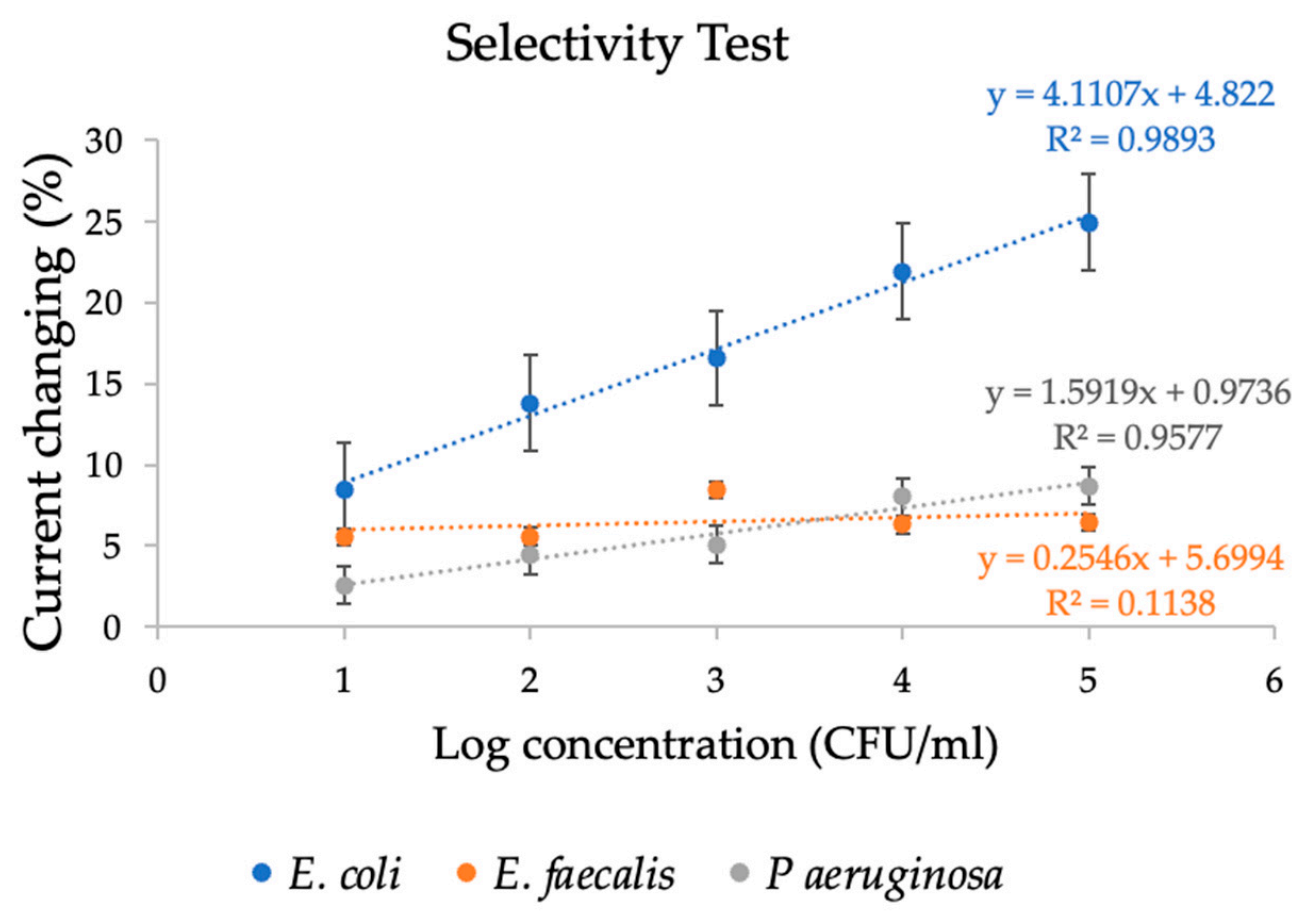
Linear comparison of the percentage current changes between *E. coli* and the non-target bacteria including *E. faecalis and P. aeruginosa*. Data are presented as mean ± SD (n = 3).

Following the selection of polymer synthesis condition 5, consisting of MAM:AAM:VP at a ratio of 2:3:1, the selectivity of the electrochemical biosensor was evaluated using two non-target bacterial species: *Enterococcus faecalis* (*E. faecalis*) *and Pseudomonas aeruginosa* (*P. aeruginosa*). The cyclic voltammograms obtained for *E. faecalis* and *P. aeruginosa* are shown in Figure 4**,** while the corresponding current responses are presented in Table 3. The linear responses obtained for *E. coli*, *E. faecalis and P. aeruginosa* are presented in Figure 5.

In Figure 5, the linear responses obtained for *E. coli, E. faecalis*, and *P. aeruginosa* exhibited slopes of 4.1107, 0.2546 and 1.5919 respectively, with corresponding R^2^ values of 0.9893, 0.1138, and 0.9577 respectively. The results demonstrated differences in the slopes and linearity of the electrochemical responses among the three bacterial species. The sensor exhibited the most favorable response toward *E. coli*, while lower responses were observed for *E. faecalis* and *P. aeruginosa*. These results suggest that the polymer synthesized under Condition 5 provided preferential recognition of *E. coli*, supporting its potential for selective detection of the target bacterium. However, the response toward *E. faecalis* showed poor linearity, as indicated by the low R^2^ value of 0.1138. Therefore, the slope for *E. faecalis* should be interpreted with caution and should not be considered alone as evidence of a concentration-dependent response. In contrast, the responses for *E. coli* and *P. aeruginosa* showed relatively good linearity, with R^2^ values of 0.9893 and 0.9577, respectively. The differences in response may be associated with differences in bacterial cell characteristics, including cell size, surface chemistry, and cell-envelope structure. In particular, *E. coli* and *P. aeruginosa* are Gram-negative bacteria, whereas *E. faecalis* is Gram-positive. Differences in cell-envelope composition and surface functional groups may influence the interaction between bacterial cells and the MIP-modified electrode and consequently affect the observed electrochemical response. However, these factors were not independently investigated in the present study and therefore cannot be considered definitive mechanisms. Overall, the lower relative responses observed for the non-target bacteria, together with the strong linear response obtained for *E. coli*, indicate a preferential response toward the target bacterium under the investigated conditions. However, the poor linearity observed for *E. faecalis* should be considered when interpreting its relative response. Further validation using additional bacterial species, standardized bacterial concentrations, and appropriate control experiments would be required to further establish the selectivity of the proposed biosensor.

**Figure 6:**
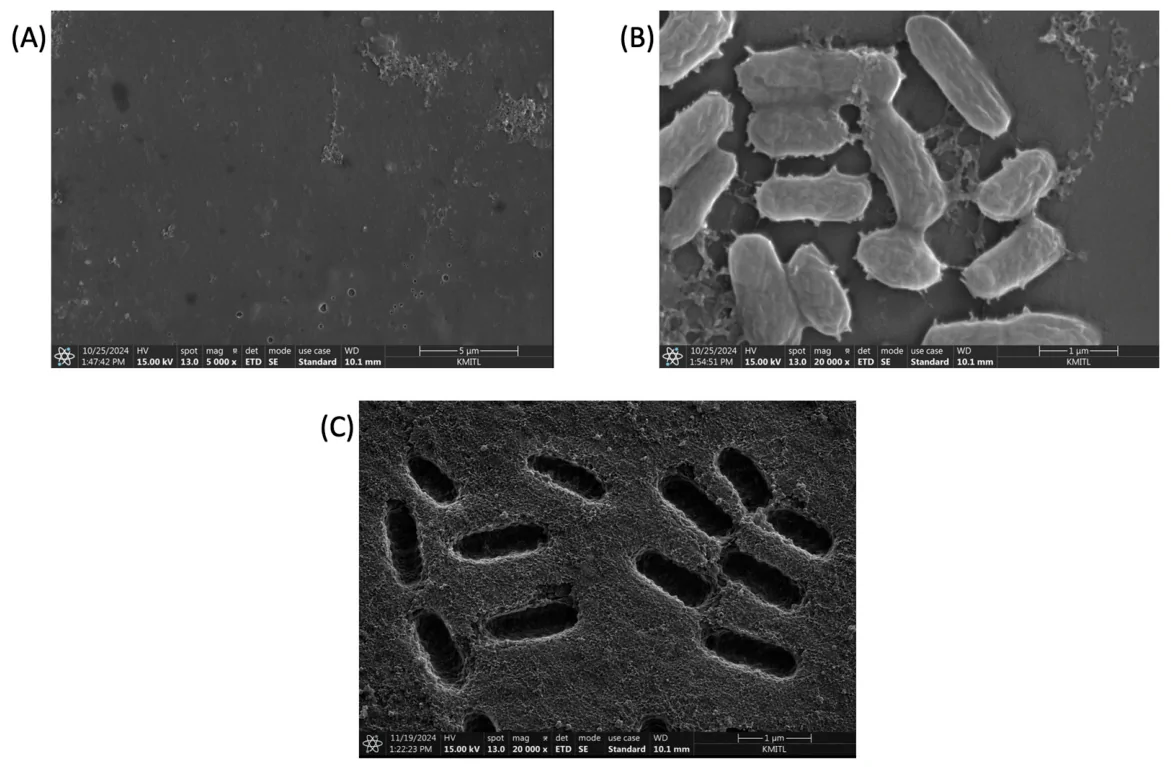
SEM images of (**A**) the polymer surface before imprinting, (**B**) the *E. coli* -imprinted polymer with *E. coli* templates on the surface, and (**C**) the polymer after *E. coli* template removal, showing cavity-like structures corresponding to the morphology of *E. coli*.

Scanning Electron Microscopy (SEM) images were used to characterize the morphological changes in the polymer during the imprinting process. Figure 6 shows (A) the polymer surface before imprinting and (B) the bacteria-imprinted polymer with *E. coli* templates on the polymer surface. After template removal, cavity-like features became visible on the polymer surface (Figure 6C), indicating the formation of vacant spaces previously occupied by the *E coli* cells. These surface cavities showed an elongated morphology generally corresponding to the rod-shaped structure of *E. coli*, consistent with bacterial template removal and the formation of potential recognition sites within the polymer matrix.

The calculated limit of detection (LOD) and limit of quantification (LOQ) values were obtained from the corresponding calibration curve for E. coli detection. The LOD was calculated using the equation (LOD = 3σ/Slope), while the LOQ was calculated using (LOQ = 10σ/Slope), where σ is the standard deviation. For *E. coli*, the calculated LOD and LOQ were 1.52 and 3.58 CFU/mL, respectively. The LOQ was higher than the LOD, as expected, reflecting the more stringent statistical criterion required for reliable quantification compared with detection. It should be noted that the calculated LOD and LOQ values were below the lowest experimentally evaluated concentration of 10 CFU/mL. Therefore, these values represent calculated estimates derived from the calibration curve and were not experimentally validated at concentrations below 10 CFU/mL.

## 4. Discussion

The higher electrochemical response observed for the MAM:AAM:VP formulation at a ratio of 2:3:1 may be associated with a favorable balance between the chemical functionality and structural properties of the imprinted polymer network. MAM, AAM, and VP provide different functional groups that may contribute to non-covalent interactions with components on the *E. coli* surface. MAM and AAM contain amide functionalities that may participate in hydrogen-bonding interactions, while VP contains a lactam carbonyl group that may provide an additional hydrogen-bond acceptor site. Therefore, the combined presence of these functional groups may contribute to the observed response toward *E. coli*.

The relatively high proportion of AAM in the 2:3:1 formulation may provide a greater availability of polar functional groups, while the incorporation of MAM and VP may contribute additional chemical functionality and influence the local polymer environment. These compositional differences may affect the formation, accessibility, and chemical environment of the imprinted cavities. The higher response observed for Condition 5 may reflect a combination of functional-group availability and polymer-network characteristics rather than a single specific molecular interaction. The enhanced response toward *E. coli* may involve multiple non-covalent interactions between the polymer and bacterial surface components. *E. coli* possesses a Gram-negative outer membrane containing lipopolysaccharides (LPS), phospholipids, and surface-associated proteins, which provide a variety of potential interaction sites with the polymer. The amide and lactam carbonyl functionalities in the 2:3:1 formulation may contribute to these interactions. Nevertheless, the observed response should not be attributed to a single specific molecular interaction, as the present study did not directly determine the contribution of individual interactions.

In addition, complementary chemical characterization would be useful to further evaluate the interactions between the MIP and *E. coli* and to verify the effective removal of the bacterial template after the washing step. In the present study, these interactions and template removal were not directly characterized using complementary chemical techniques. Therefore, the proposed hydrogen-bonding and dipole–dipole interactions should be considered possible mechanisms rather than experimentally confirmed interactions. Further characterization using techniques such as FTIR or other surface-sensitive analytical methods could provide additional evidence of the chemical interactions and template removal.

Differences in monomer composition may also influence the formation and accessibility of the recognition cavities. Formulations with different proportions of individual monomers may produce polymer networks with different chemical environments and structural characteristics, which could affect bacterial recognition. Thus, the higher response observed for the MAM:AAM:VP ratio of 2:3:1 may reflect a favorable combination of functional-group availability, cavity accessibility, and polymer-network characteristics under the investigated conditions. To further assess the analytical performance of the proposed sensor, its performance was compared with representative recently reported electrochemical/MIP-based bacterial sensors, as summarized in Table 4.

Previous studies have employed different monomers and polymer systems, including dopamine-based polydopamine MIP layers and other MIP compositions. For example, Idil et al. reported a whole-cell MIP sensor based on N-methacryloyl-L-histidine methyl ester (MAH), 2-hydroxyethyl methacrylate (HEMA), and ethylene glycol dimethacrylate (EGDMA) with a detection range of 10^2^–10^7^ CFU/mL and an LOD of 70 CFU/mL [50]. A dopamine-based bacteria-imprinted polydopamine sensor using a modified glassy carbon electrode achieved a detection range of 5–10^7^ CFU/mL with an LOD of 1.5 CFU/mL [51]. Another dopamine-based MIP paper device reported a detection range of 25–1200 CFU/mL and an LOD of 25 CFU/mL [52]. Another dopamine-based PDA-MIP sensor employing a bipolar electrode and Au@MOF modification with electrochemiluminescence (ECL) detection achieved a linear detection range of 1–10^6^ CFU/mL with an LOD of 1 CFU/mL [53]. In comparison, the present study employed MAM, AAM, and VP at an optimized ratio of 2:3:1 and achieved a linear detection range of 10–10^5^ CFU/mL with a calculated LOD of 1.52 CFU/mL using a disposable screen-printed electrode and CV with a ferri/ferrocyanide redox probe. These results demonstrate comparable analytical performance while providing an alternative polymer composition and a relatively simple electrochemical configuration.

In this study, the sensor response was defined as the change in electrochemical signal associated with increasing bacterial concentration. The sensitivity was evaluated from the slope of the calibration curve, where a higher slope indicates a greater change in electrochemical response per unit increase in bacterial concentration. For the optimized MAM:AAM ratio of 2:3:1, the sensor exhibited a sensitivity of 4.1107 with an R^2^ value of 0.9893 for *E. coli*. For the non-target bacteria, *E. faecalis* and *P. aeruginosa*, the corresponding slopes were 0.2546 (R^2^ = 0.1138) and 1.5919 (R^2^ = 0.9577), respectively, indicating lower response toward the non-target bacteria under the investigated conditions. The selectivity was therefore evaluated by comparing the response of the sensor toward the target *E. coli* with that toward non-target bacteria. The comparison includes detection range, sensitivity, selectivity, analysis time, and practical limitations. The proposed sensor showed a detection range of 10–10^5^ CFU/mL and a detection limit of 1.52 CFU/mL for *E. coli*. However, direct comparison among different studies should be interpreted with caution because analytical performance can be affected by differences in electrode configuration, MIP composition, electrochemical technique, sample preparation, and measurement conditions.

Overall, these results indicate that the monomer composition influences the electrochemical response of the MIP-based sensor. The 2:3:1 MAM:AAM formulation produced the highest response toward *E. coli* among the tested formulations, suggesting that this composition provided a favorable chemical and structural environment for *E. coli* recognition under the investigated conditions. Further studies using complementary characterization techniques would be required to directly confirm the proposed molecular interactions and clarify the relationship between monomer composition, cavity structure, and bacterial recognition.

## 5. Conclusions

The present study developed a molecularly imprinted polymer-based electrochemical sensor for the detection of *Escherichia coli*. The primary objective of this study was to investigate and optimize the monomer composition to identify a suitable formulation for *E. coli* recognition under controlled laboratory conditions. Optimization of the monomer composition demonstrated that the MAM:AAM:VP ratio of 2:3:1 provided the most favorable sensing performance among the investigated formulations. The enhanced response of this composition may be associated with the appropriate balance of functional groups and polymer network characteristics, which may promote the formation of accessible and chemically complementary recognition sites for *E. coli*. The optimized MIP electrode also exhibited a greater electrochemical response toward *E. coli* than toward the non-target bacteria, *E. faecalis* and *P. aeruginosa*, indicating its preferential recognition capability under the tested conditions. Furthermore, the optimized sensor showed a strong concentration-dependent response, demonstrating its potential for selective *E. coli* detection. However, the present study was limited to evaluations under controlled laboratory conditions and did not include validation using real samples. Further investigation using real sample matrices will be necessary to assess matrix effects, recovery, reproducibility and the practical applicability of the developed sensor. In addition, further studies comparing the performance of the MIP-based sensor with the corresponding non-imprinted polymer sensor in real sample matrices would provide additional evidence of the molecular imprinting effect and the specificity of the developed sensor. Overall, these findings highlight the importance of monomer composition in controlling the recognition performance of MIP-based electrochemical sensors and provide a basis for further development of the platform toward practical bacterial detection.

## Figures and Tables

**Table 1:** Compositions of the six polymer formulations used for *E.coli* MIP synthesis.

Condition	Molar Ratio (mol:mol:mol)	MAM (mg)	AAM (mg)	VP (µL)
1	1:1:1	8.5	7.1	10.7
2	2:1:1	17.0	7.1	10.7
3	1:2:1	8.5	14.2	10.7
4	3:2:1	25.5	14.2	10.7
5	2:3:1	17.0	21.4	10.7
6	3:3:1	25.5	21.4	10.7

**Table 2:** Current responses obtained under six different polymer synthesis conditions.

**Condition 1**	**Current (µA)**	**∆I (µA)**	**% Current change**
Blank	44.19		
10 CFU/mL	39.05	5.13	11.61
100 CFU/mL	38.37	5.82	13.17
1000 CFU/mL	38.72	5.47	12.37
10,000 CFU/mL	38.91	5.28	11.94
100,000 CFU/mL	34.24	9.95	22.52
**Condition 2**	**Current (µA)**	**∆I (µA)**	**% Current change**
Blank	45.85		
10 CFU/mL	40.80	5.05	11.01
100 CFU/mL	40.44	5.40	11.79
1000 CFU/mL	40.84	5.01	10.92
10,000 CFU/mL	40.16	5.68	12.40
100,000 CFU/mL	36.16	9.69	21.13
**Condition 3**	**Current (µA)**	**∆I (µA)**	**% Current change**
Blank	50.69		
10 CFU/mL	42.73	7.96	15.70
100 CFU/mL	43.44	7.25	14.30
1000 CFU/mL	42.95	7.75	15.28
10,000 CFU/mL	42.15	8.55	16.86
100,000 CFU/mL	37.60	13.09	25.82
**Condition 4**	**Current (µA)**	**∆I (µA)**	**% Current change**
Blank	39.55		
10 CFU/mL	36.61	2.94	7.42
100 CFU/mL	37.16	2.39	6.05
1000 CFU/mL	37.25	2.30	5.83
10,000 CFU/mL	36.52	3.03	7.65
100,000 CFU/mL	32.36	7.19	18.18
**Condition 5**	**Current (µA)**	**∆I (µA)**	**% Current change**
Blank	42.32		
10 CFU/mL	38.73	3.59	8.48
100 CFU/mL	36.47	5.85	13.83
1000 CFU/mL	35.32	7.01	16.55
10,000 CFU/mL	33.03	9.29	21.95
100,000 CFU/mL	31.76	10.57	24.97
**Condition 6**	**Current (µA)**	**∆I (µA)**	**% Current change**
Blank	41.35		
10 CFU/mL	39.93	1.42	3.43
100 CFU/mL	39.27	2.08	5.03
1000 CFU/mL	39.16	2.20	5.31
10,000 CFU/mL	38.46	2.89	6.99
100,000 CFU/mL	34.70	6.65	16.09

**Table 3:** Current responses obtained for the non-target bacteria, *E. faecalis* and *P. aeruginosa*.

***E. faecalis***	**Current (µA)**	**∆I (µA)**	**% Current change**
Blank	30.64		
10 CFU/mL	28.95	1.70	5.54
100 CFU/mL	28.94	1.71	5.57
1000 CFU/mL	28.05	2.59	8.47
10,000 CFU/mL	28.71	1.93	6.30
100,000 CFU/mL	28.67	1.98	6.45
***P. aeruginosa***	**Current (µA)**	**∆I (µA)**	**% Current chang** **e**
Blank	29.02		
10 CFU/mL	28.28	0.74	2.54
100 CFU/mL	27.73	1.28	4.43
1000 CFU/mL	27.55	1.47	5.05
10,000 CFU/mL	26.68	2.34	8.05
100,000 CFU/mL	26.50	2.52	8.68

**Table 4:** Comparison of the analytical performance of MIP-based sensors for *E. coli* detection.

Sensor	Electrode Platform	Polymer/MIP	Method	Range	LOD
This work	SPE	MAM/AAM/VP-MIP	CV	10–10^5^ CFU/mL	1.52 CFU/mL
[50]	Au electrode	Whole-cell MIP	Capacitive	10^2^–10^7^ CFU/mL	70 CFU/mL
[51]	GCE/SPE	Polydopamine BIP	SWV/CV/EIS	5–10^7^ CFU/mL	1.5 CFU/mL
[52]	Paper device	Dopamine-MIP	Colorimetric	25–1200 CFU/mL	25 CFU/mL
[53]	Bipolar electrode	PDA-MIP + Au@MOF	ECL	1–10^6^ CFU/mL	1 CFU/mL

## Data Availability

The original contributions presented in this study are included in the article. Further inquiries can be directed to the corresponding author.
